# Supplementary material for: Non-coding autoimmune risk variant defines role for ICOS in T peripheral helper cell development
Source: Nat Commun. 2024 Mar 9;15:2150. doi: 10.1038/s41467-024-46457-8 (PMC10923805; doi:10.1038/s41467-024-46457-8)
Supplement: Supplementary file 1 — Supplementary Information [file 41467_2024_46457_MOESM1_ESM.pdf]

Supplementary Figures

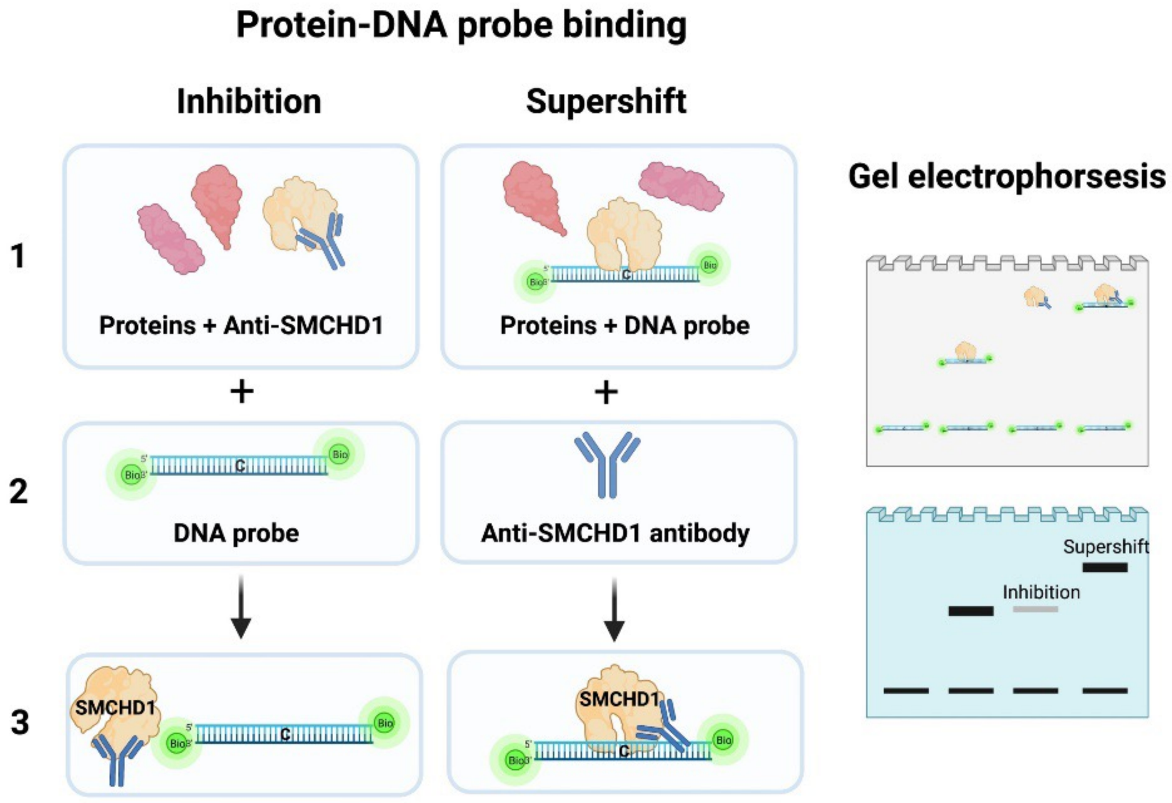

Supplementary Figure 1. **Differential effects of antibodies during EMSA in Figure 1B**. Pre-incubation of anti-SMCHD1 with nuclear extract inhibits DNA probe binding, reducing the amount of SMCHD1-DNA complex observed on the EMSA gel image. Incubation with anti-SMCHD1 after nuclear extract/DNA probe complexes have already been formed results in supershifted complexes of SMCHD1, anti-SMCHD1, and DNA probe. This illustration was created with BioRender.com.

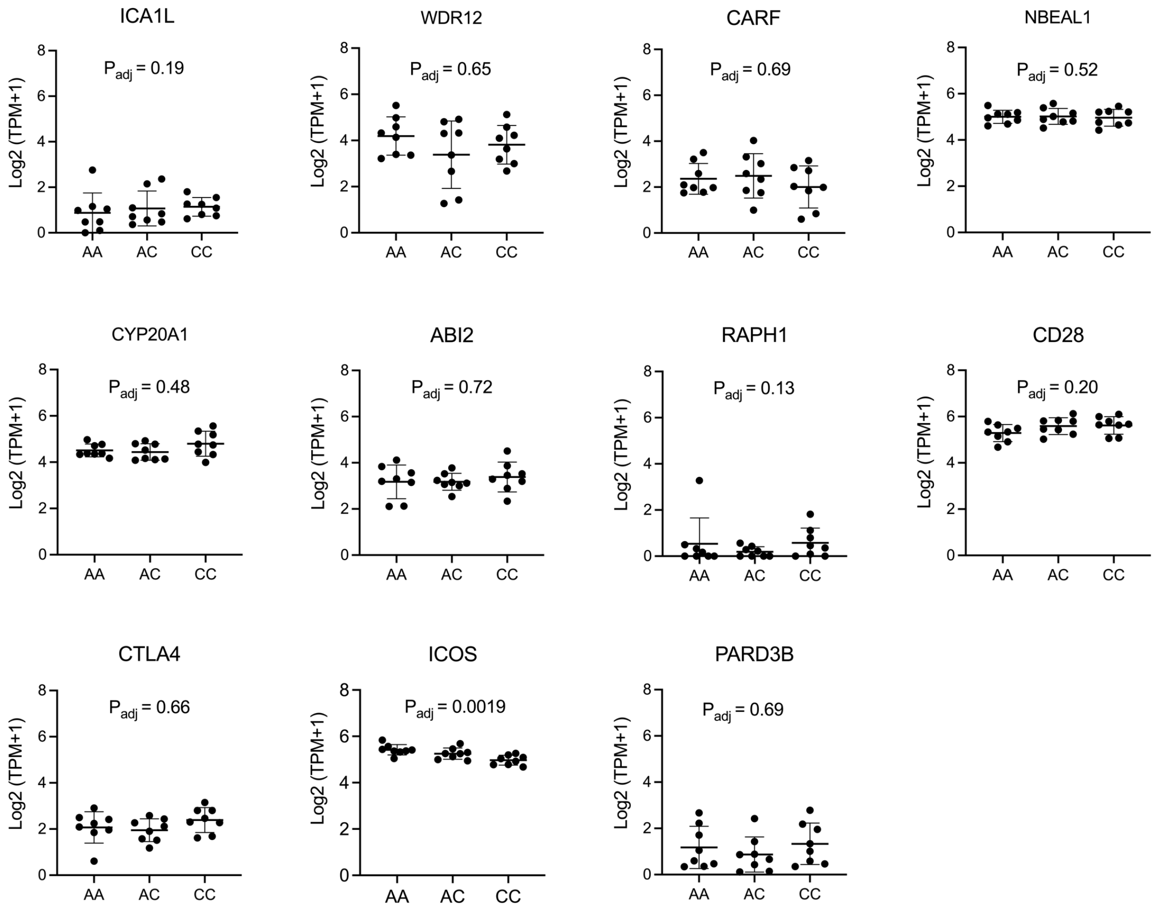

Supplementary Figure 2. **cis-eQTL mapping analysis** was performed using low-input RNA sequencing in resting CD4+ T cells from 24 healthy donors (8 A/A, 8 A/C, and 8 C/C genotypes at rs117701653). We examined 11 protein coding genes that have a transcription start site (TSS) within a 1 MB window of SNP rs117701653, namely *ICA1L*, *WDR12*, *CARF*, *NBEAL1*, *CYP20A1*, *ABI2*, *RAPH1*, *CD28*, *CTLA4*, *ICOS*, and *PARD3B*. Expression levels were corrected for age and sex and residuals were rank-normal transformed. P values from a linear model implemented in QTLtools were shown. *P* values were computed using a linear model by QTLtools for association between genotypes and expression levels corrected for multiple comparison, age, sex, and rank-normal transformed residuals.

A Wild type Jurkat cells

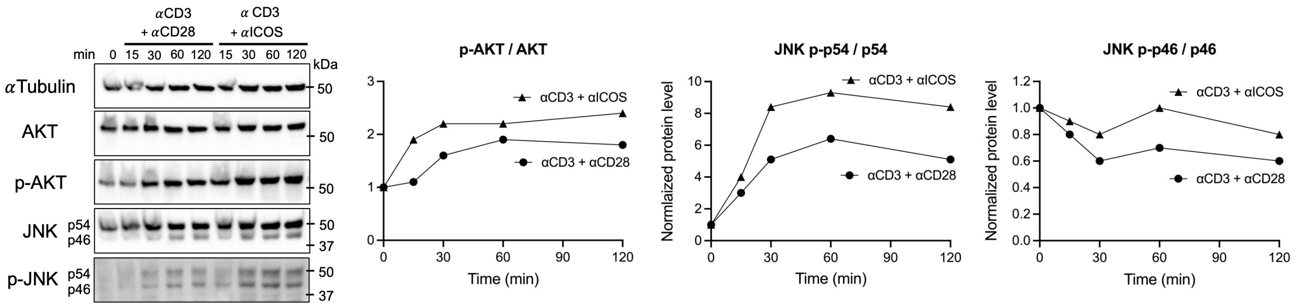

B Base-edited Jurkat clones

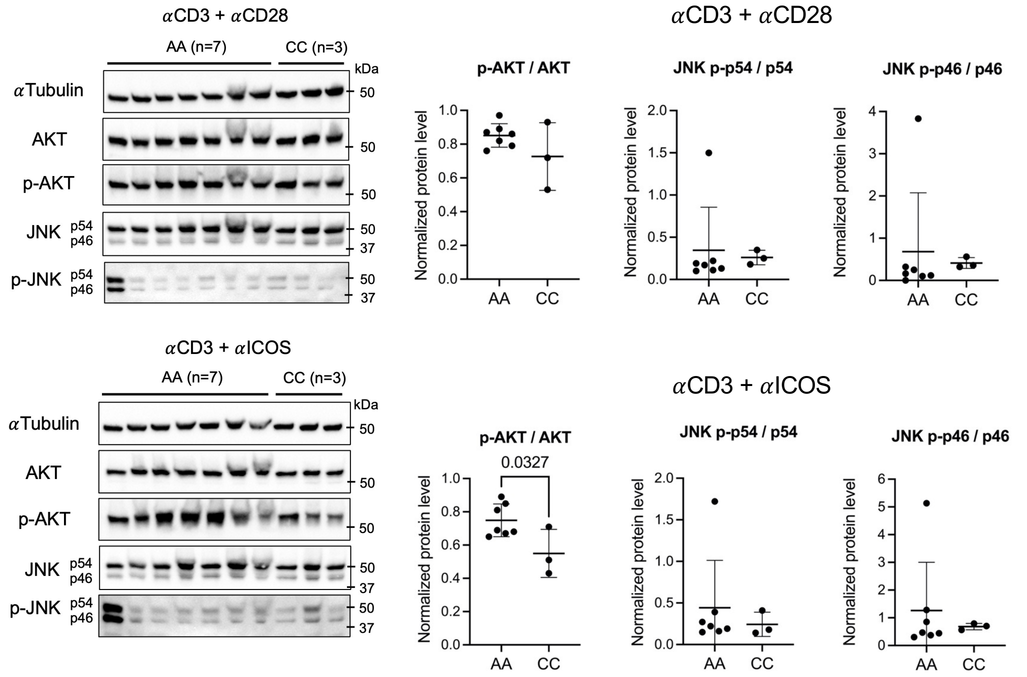

Supplementary Figure 3. **Genotype at s117701653 controls ICOS signaling.** (**A**) In wild-type Jurkat cells stimulated with either anti-CD3/CD28 or anti-CD3/ICOS antibodies over different time periods, the phosphorylated levels of AKT and JNK were examined once using western blot analysis. Protein expressions were quantified by measuring the band intensity corresponding to α-Tubulin, AKT, and JNK. The signaling activity was assessed by examining the relative expression of phospho-AKT, phospho-JNK p54 and p46 compared to their total expressions. (**B**) AA (n=7) and CC (n=3) CRISPR-edited Jurkat clones were stimulated with either anti-CD3/CD28 or anti-CD3/ICOS for 30 minutes. Then, the signaling activities as indicated by p-AKT/AKT, JNK p-p54/JNK and JNK p-p46 were tested once to compare between the AA and CC clones. *P* value from unpaired t-test with two-tailed significance. AKT: AKT serine/threonine kinase, JNK: c-Jun N-terminal kinases. Uncropped western blotting blots are included in the Source Data file.

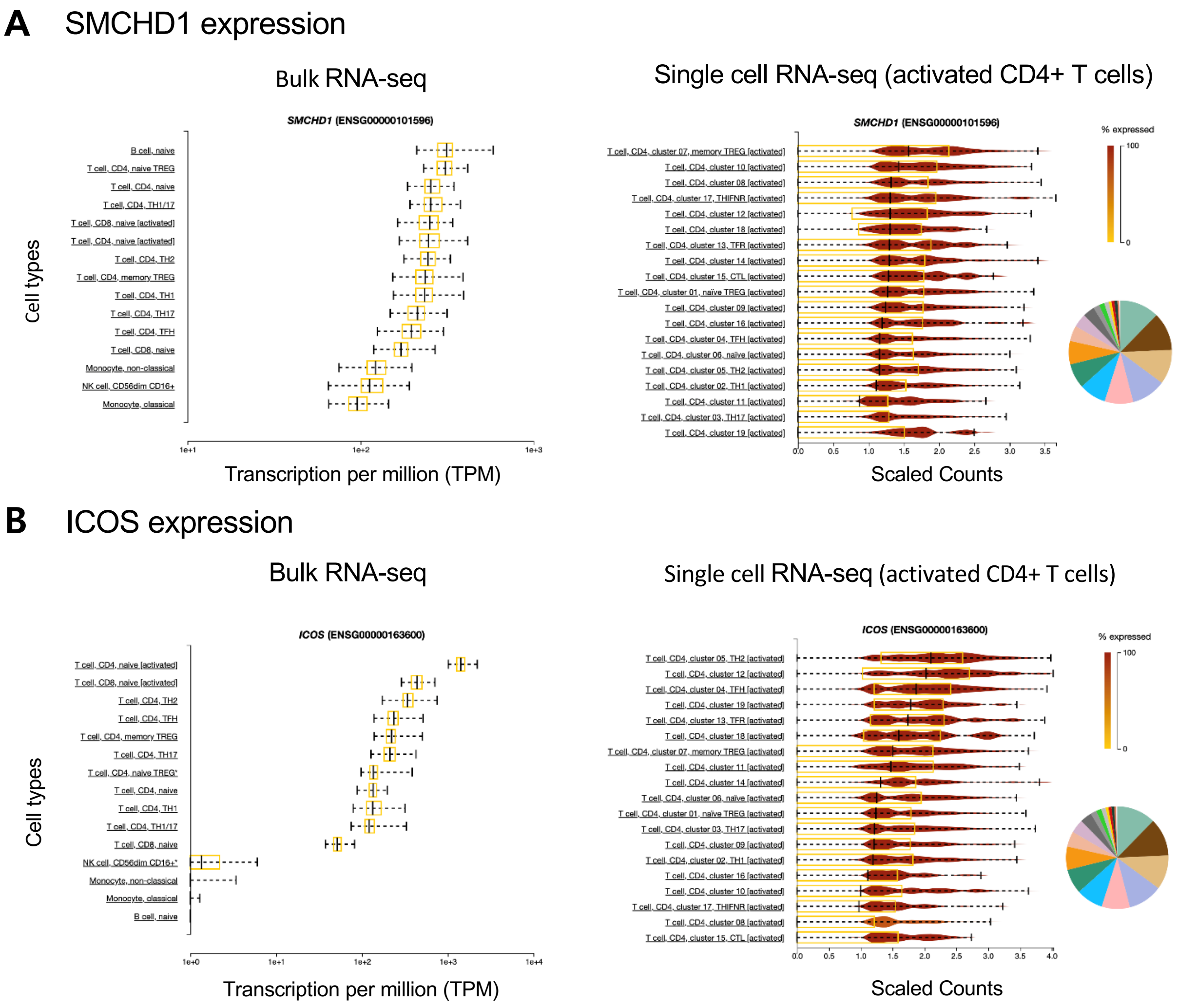

Supplementary Figure 4. (A) SMCHD1 and (B) ICOS expression by bulk RNA sequencing and single cell RNA sequencing were retrieved from https://dice-database.org/landing. Bulk RNA-seq; X-axis represents expression level of depicted genes in transcripts per million (TPM). Boxes indicate 25-75 % interquartile ranges, and whiskers indicate minimum to maximum. Single cell RNA-seq; violin plots display scaled counts of expression levels in each cell cluster. The color scale indicates the fraction of cells that express the depicted genes within each cluster of activated CD4+ T cells. Boxes indicate 25-75 % interquartile ranges, and whiskers indicate minimum to maximum.

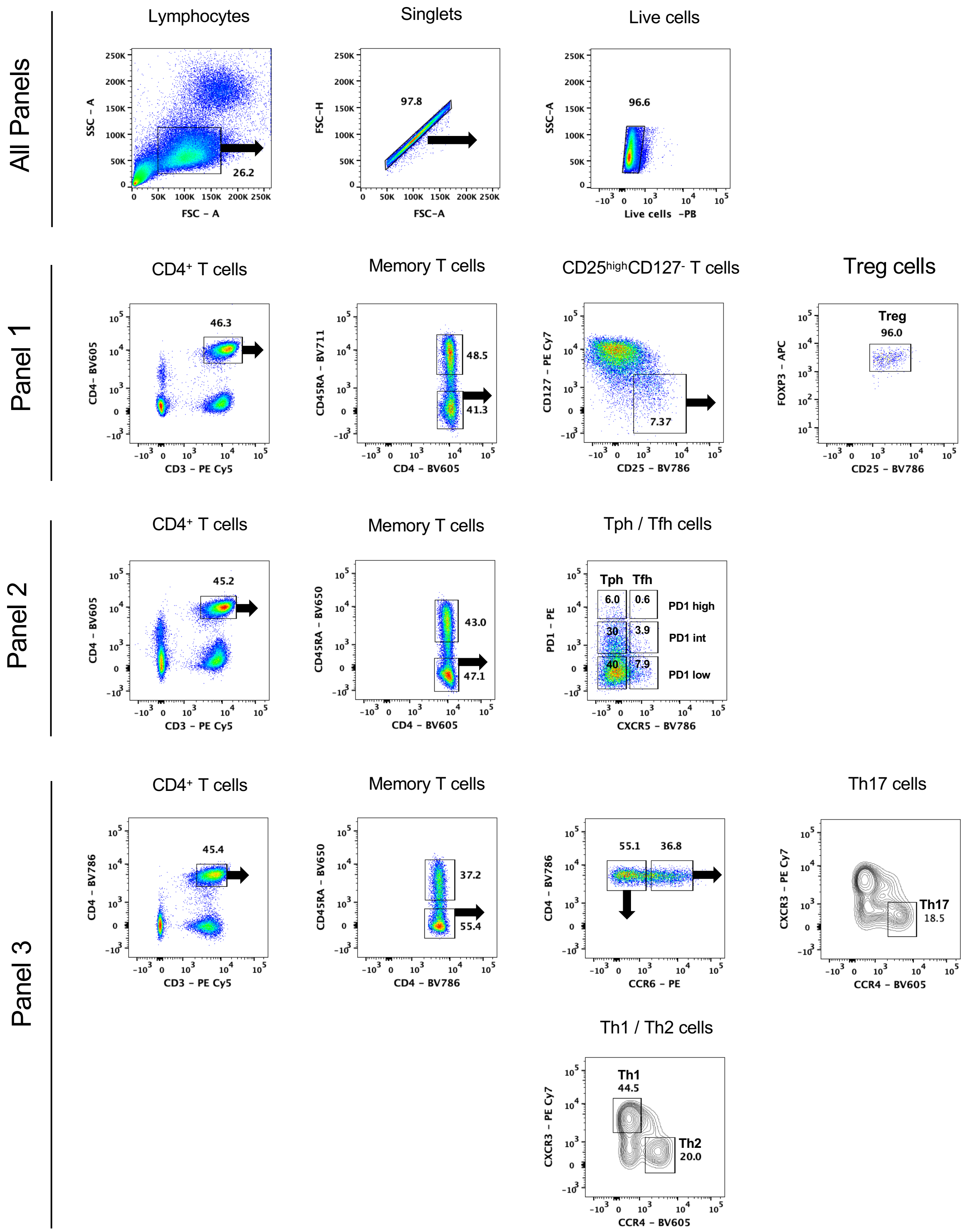

Supplementary Figure 5. Gating strategies to identify $CD3^+CD4^+CD25^{high}CD127^-FOXP3^+$ Treg cells (panel 1); $CD3^+CD4^+CD45RA^-CXCR5^-PD\text{-}1^{high}$ Tph cells and $CD3^+CD4^+CD45RA^-CXCR5^+PD\text{-}1^{high}$ Tfh cells (panel 2); $CD3^+CD4^+CD45RA^-$ $CCR6^-CXCR3^+CCR4^-$ Th1 cells, $CD3^+CD4^+CD45RA^-CCR6^-CXCR3^-CCR4^+$ Th2 cells, and $CD3^+CD4^+CD45RA^-CCR6^+CXCR3^-CCR4^+$ Th17 cells (panel 3).

ICOS expression by flow cytometry

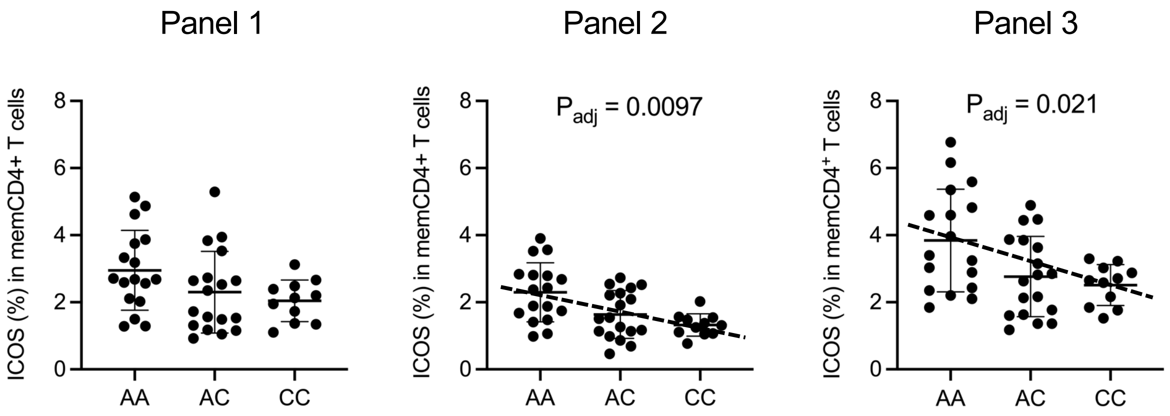

Supplementary Figure 6. **Association of rs117701653 genotype with frequency of ICOS+ cells among memory CD4+ T cells.** The frequency of ICOS expression was determined by flow cytometry for 46 healthy subjects with A/A (n = 17), A/C (n = 18), and C/C (n = 11) genotype at SNP rs117701653 by flow cytometry analysis, as per antibody panels depicted in Figure S5. Error bars are represented by Mean ± S.D. *P* values were determined using a linear regression model adjusted for age and sex.

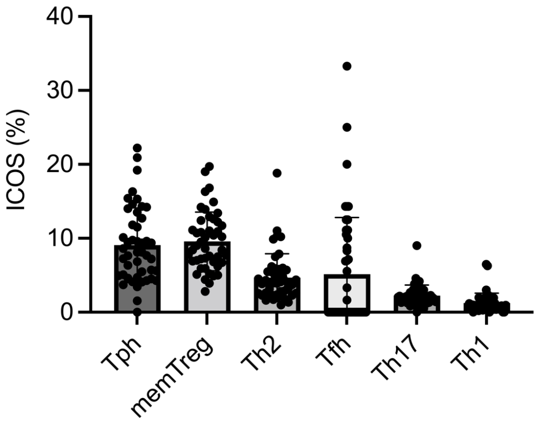

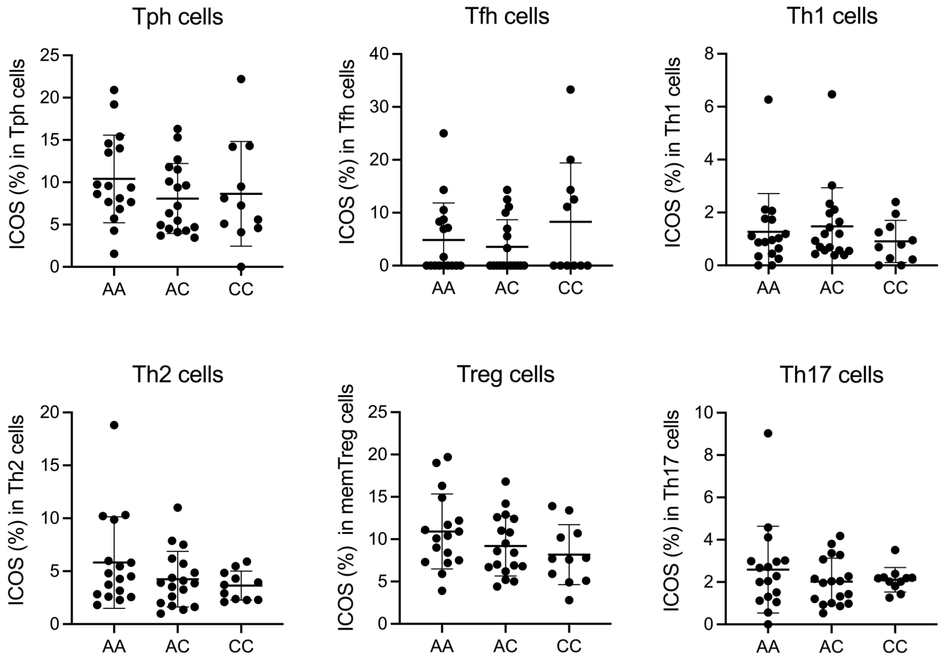

Supplementary Figure 7. **Frequency of CD4^{+} T cell subsets (Tph, memory Treg, Th2, Tfh, Th17, Th1) that express ICOS** was determined by flow cytometry for healthy subjects bearing A/A (n = 17), A/C (n = 18), and C/C (n = 11) genotype at SNP rs117701653.

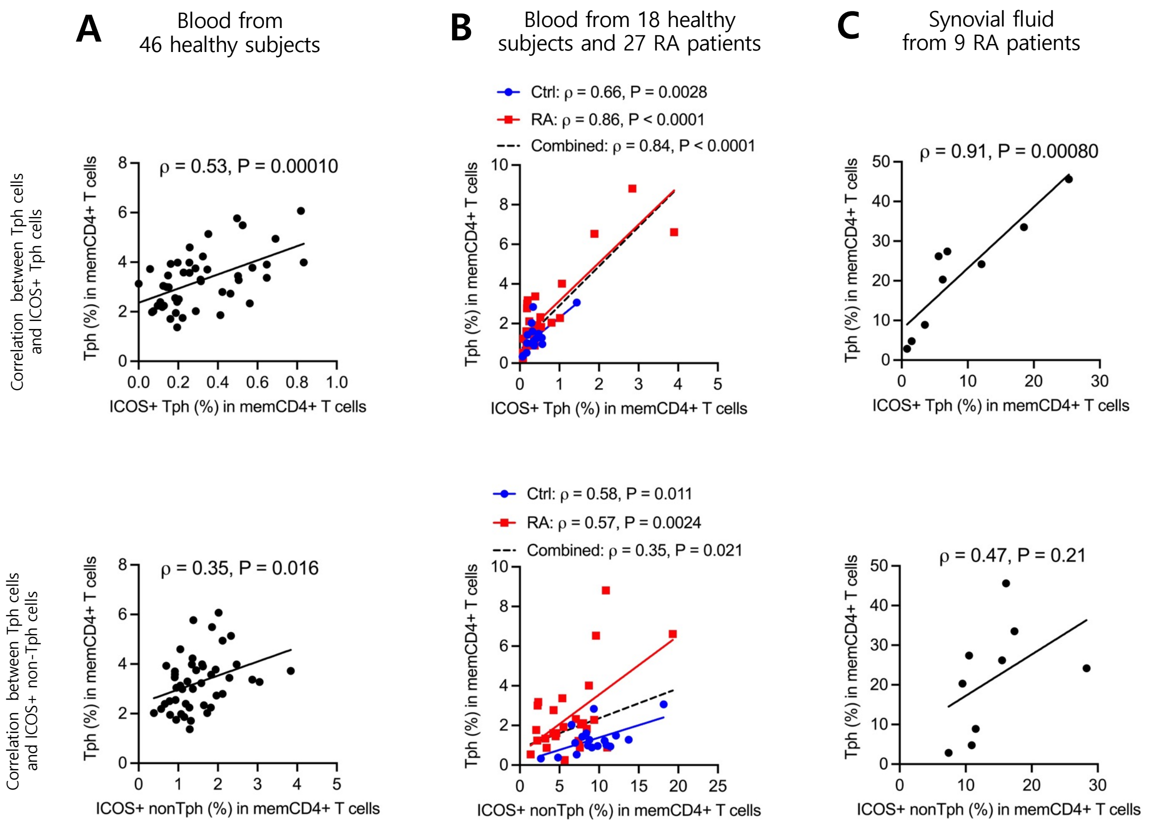

Supplementary Figure 8. **Frequency of ICOS⁺ Tph or ICOS⁺ non-Tph cells in memory CD4⁺ T cells correlates with frequency of Tph cells in memory CD4⁺ T cells**. The proportion of subsets depicted was determined by (**A**) flow cytometry with PBMCs from 46 healthy subjects, (**B**) mass cytometry with PBMCs from 18 healthy controls (colored in blue) and 27 RA patients (colored in red), and (**C**) flow cytometry with synovial fluid from 9 RA patients. Pearson correlation coefficients and *p* values with a two-tailed test were used to measure linear correlations.

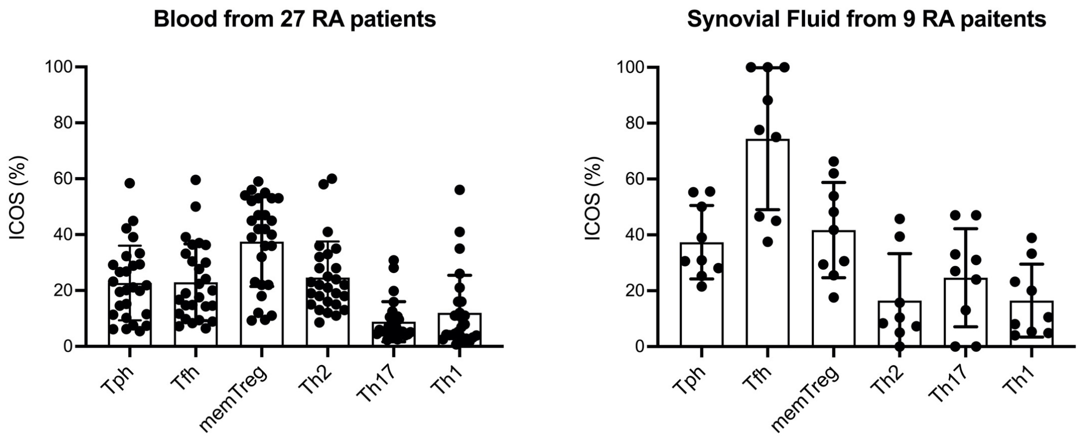

Supplementary Figure 9. **Frequency of ICOS+ cells within CD4+ T cell subpopulations in RA** (Tph, Tfh, memory Treg, Th2, Th17, and Th1) in blood from 27 patients and synovial fluid from 9 patients.

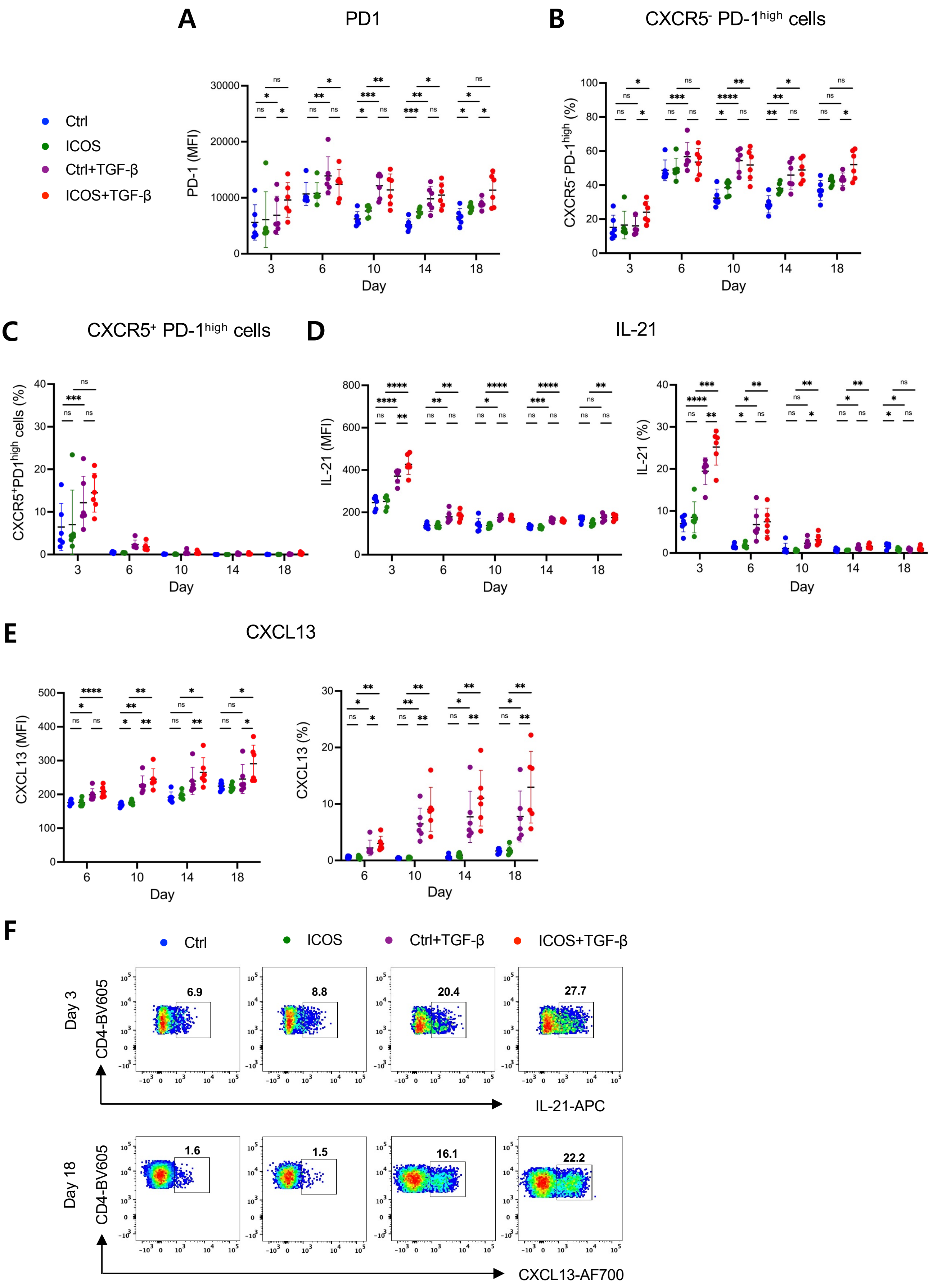

A
PD1
B
CXCR5- PD-1high cells
Ctrl
ICOS
Ctrl+TGF-β
ICOS+TGF-β
PD-1 (MFI)
CXCR5- PD-1high (%)
Day
C
CXCR5+ PD-1high cells
CXCR5+PD1high cells (%)
D
IL-21
IL-21 (MFI)
IL-21 (%)
E
CXCL13
CXCL13 (MFI)
CXCL13 (%)
F
Day 3
Day 18
CD4-BV605
IL-21-APC
CXCL13-AF700
6.9
8.8
20.4
27.7
1.6
1.5
16.1
22.2

Supplementary Figure 10. ICOS stimulation accelerates the development of TGF-β-induced CXCR5^{-}PD-1^{high} Tph-like cells expressing IL-21 and CXCL13. Human memory CD4^{+} T cells from 6 healthy subjects were differentiated with anti-CD3/CD28 stimulation in the indicated combination of TGF-β and anti-ICOS stimulation for varying period. Control (Blue), ICOS (Green), TGF-β (Purple), TGF-β+ICOS (Red) stimulation groups were evaluated in time course. (**A**) MFI of PD-1 in whole population. (**B**) Frequency of CXCR5^{-} PD-1^{high} cells. (**C**) Frequency of CXCR5^{+} PD-1^{high} cells. MFI of (**D**) IL-21 and (**E**) CXCL13 in whole population. (**F**) Representative dot plots of IL-21 and CXCL13. MFI, mean fluorescence intensity. Error bars are expressed as mean $\pm$ S.D. **P* < 0.05, ***P* < 0.01, ****P* < 0.001, *****P* < 0.0001. *P* values were determined using one-way ANOVA followed by uncorrected Fisher's LSD test. Exact *P* values were provided in the Source Data.

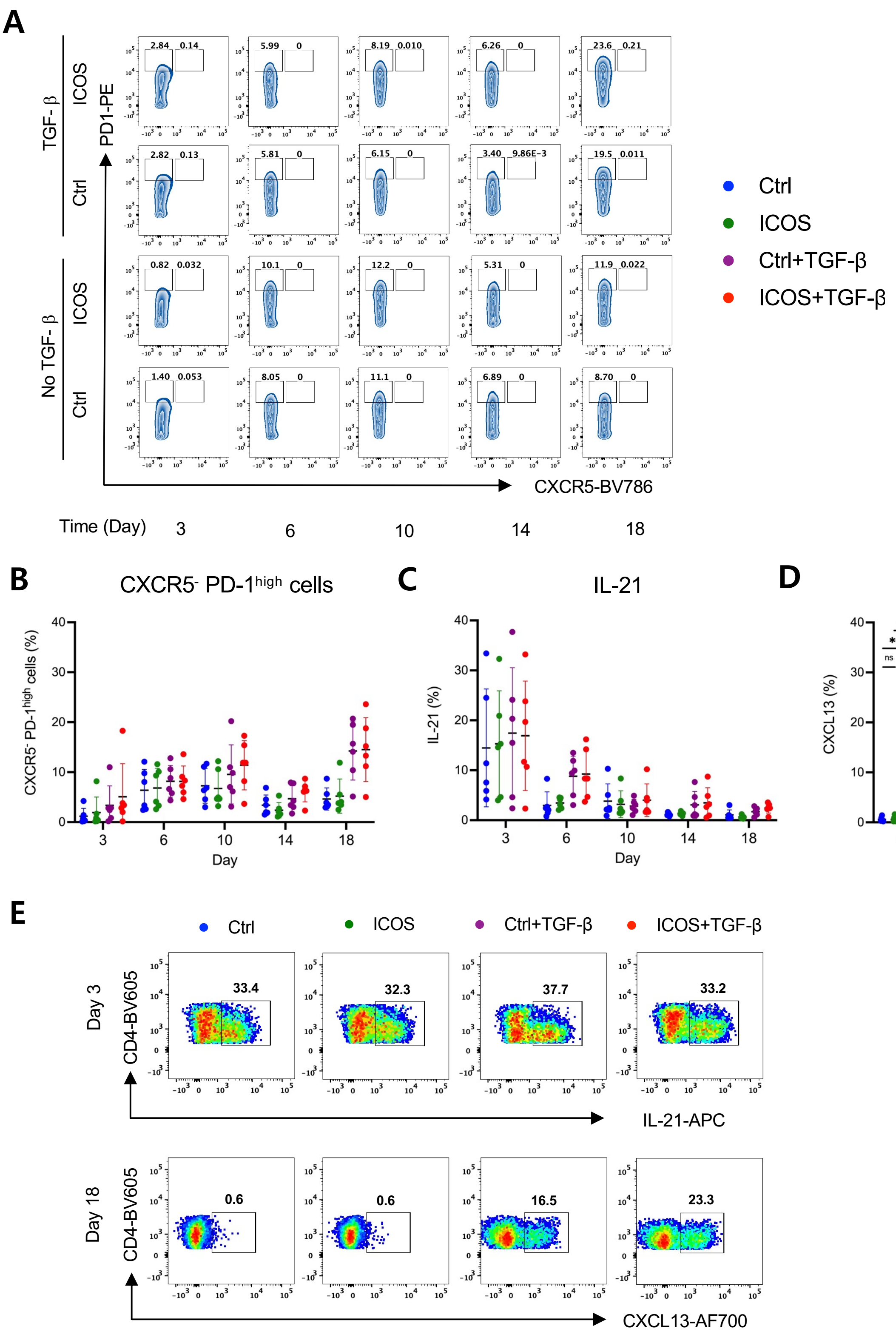
A
TGF-β
ICOS
Ctrl
No TGF-β
ICOS
Ctrl
PD1-PE
CXCR5-BV786
Time (Day) 3 6 10 14 18
2.84 0.14
5.99 0
8.19 0.010
6.26 0
23.6 0.21
2.82 0.13
5.81 0
6.15 0
3.40 9.86E-3
19.5 0.011
0.82 0.032
10.1 0
12.2 0
5.31 0
11.9 0.022
1.40 0.053
8.05 0
11.1 0
6.89 0
8.70 0
Ctrl
ICOS
Ctrl+TGF-β
ICOS+TGF-β
B
CXCR5- PD-1high cells
CXCR5- PD-1high cells (%)
Day
C
IL-21
IL-21 (%)
Day
D
CXCL13
CXCL13 (%)
Day
E
Ctrl
ICOS
Ctrl+TGF-β
ICOS+TGF-β
Day 3
CD4-BV605
33.4
32.3
37.7
33.2
IL-21-APC
Day 18
CD4-BV605
0.6
0.6
16.5
23.3
CXCL13-AF700

Supplementary Figure 11. Naive CD4+ T cells differentiating into CXCR5$^-$PD-1^{high} Tph-like cells was not influenced by ICOS stimulation. Human naive CD4+ T cells from 6 healthy donors were differentiated with anti-CD3/CD28 stimulation in the indicated combination of TGF-β and anti-ICOS stimulation for different period. Control (Blue), ICOS (Green), TGF-β (Purple), TGF-β+ICOS (Red) stimulation groups were evaluated. (**A**) Representative dot plots by flow cytometry of CXCR5 and PD-1 to gate CXCR5$^-$PD-1^{high} Tph-like cells and CXCR5$^+$PD-1^{high} Tfh-like cells. (**B**) Frequency of CXCR5$^-$ PD-1^{high} cells. Frequency of (**C**) IL-21 and (**D**) CXCL13 in whole population. (**E**) Representative dot plots of IL-21 and CXCL13. Error bars are expressed as mean ± S.D. *$P < 0.05$, **$P < 0.01$, ***$P < 0.001$, ****$P < 0.0001$. *P* values were determined using one-way ANOVA followed by uncorrected Fisher's LSD test. Exact *P* values were provided in the Source Data.

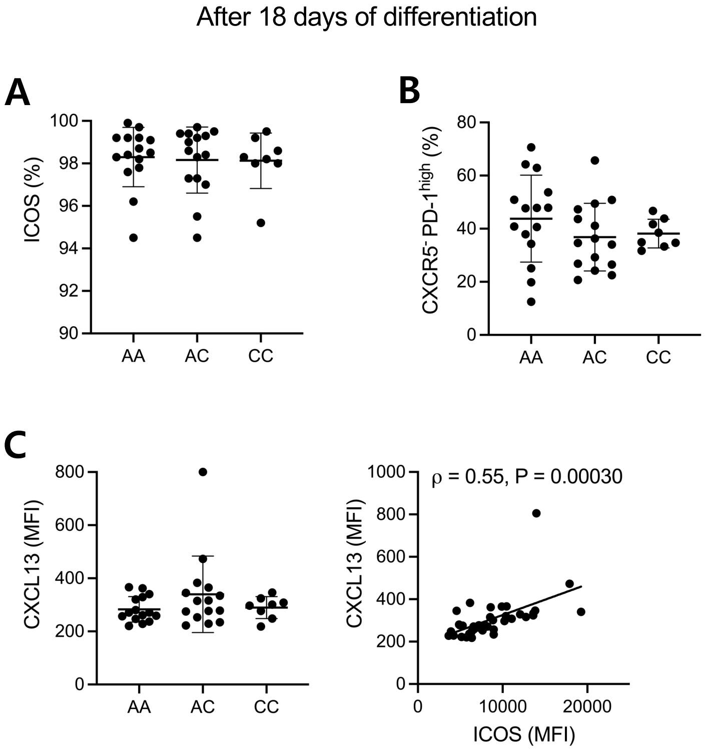

Supplementary Figure 12. Genotype at rs117701653 does not correlate with differentiation of $CXCR5^{-}PD\text{-}1^{high}$ Tph-like $CD4^{+}$ T cells at day 18. Memory $CD4^{+}$ T cells from 38 healthy donors with A/A (n = 15), A/C (n = 15), C/C (n = 8) genotype at rs117701653 SNP were differentiated using anti-CD3/CD28 bead and anti-ICOS antibody in the presence of TGF-β. After 18 days of differentiation, (**A**) frequency of $ICOS^{+}$ cells, (**B**) frequency of $CXCR5^{-}PD\text{-}1^{high}$ cells, (**C**) MFI of CXCL13 and correlation between MFI of ICOS and CXCL13 across whole individuals were displayed. MFI, mean fluorescence intensity. Error bars are expressed as mean ± S.D. Pearson correlation statistics with a two-tailed test (**C**).

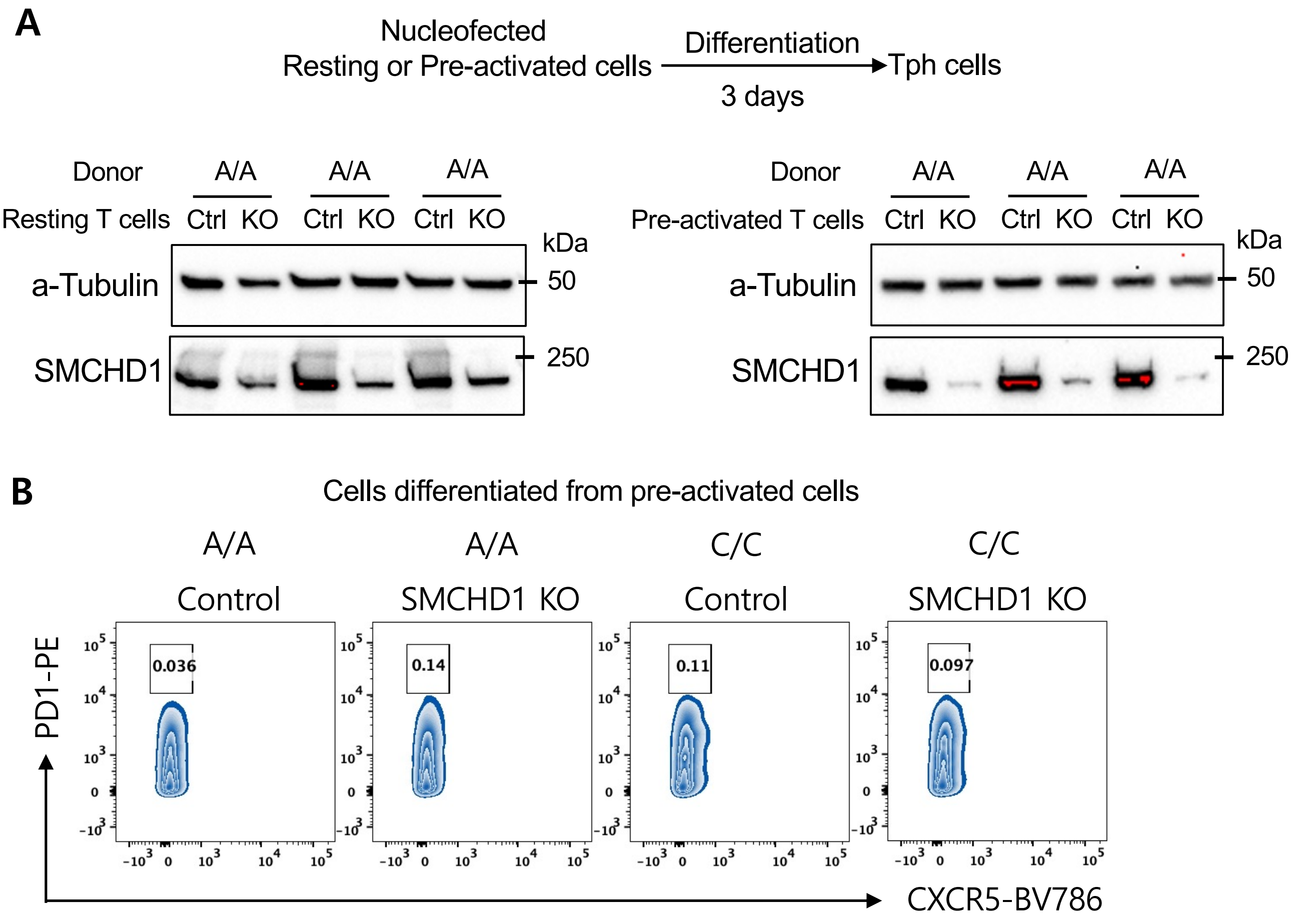

Supplementary Figure 13. SMCHD1 deletion using CRISPR-Cas9 was optimized in memory CD4+ T cells isolated from 5 healthy individuals. (**A**) Resting cells or cells preactivated with anti-CD3/CD28 for 2 days were nucleofected with Cas9 protein and either a negative control or SMCHD1-targeting sgRNA. Following this, the resting and preactivated cells were stimulated using anti-CD3/CD28, anti-ICOS, and TGF-β for 3 days. The efficiency of SMCHD1 deletion was assessed by western blot, which demonstrated the successful removal of over 90% of SMCHD1 in the cells differentiated from the preactivated cells. The SMCHD1 deletion was successfully confirmed three times with independent biological samples, including Figure 6G. (**B**) Flow cytometry gating of CXCR5- PD-1high cells revealed that the preactivation condition resulted in a lack of high expression of PD-1. Uncropped western blotting blots are included in the Source Data file.

Supplementary Tables

Supplementary Table 1. Mass spectrometry of rs117701653-bound proteins by FREP

Protein ID	Molecular weight	Bio-rs1177	Bio-rs1177 + Competitor	Bio-Ctrl
ALB	69 kDa	14	26	23
PARP1	113 kDa	14	17	26
DST	861 kDa	3	1	6
RYR1	565 kDa	3	1	2
H3BNH8	10 kDa	3	0	0
ACACB	277 kDa	3	0	1
SMCHD1	226 kDa	3	0	0
SYNE1	1011 kDa	2	0	5
DYNC1H1	532 kDa	2	1	3
GOLGB1	376 kDa	2	0	4
SF3B1	146 kDa	2	0	3
TOP1	91 kDa	1	3	1
SMG1	410 kDa	1	0	4
ROCK1	158 kDa	1	0	3
SPEN	402 kDa	1	0	3
IFI16	88 kDa	0	9	0
NONO	54 kDa	0	9	0
SFPQ	76 kDa	0	7	0
ACTB	42 kDa	0	6	0
DDX5	69 kDa	0	4	0
HNRNPM	78 kDa	0	4	2
PUF60	60 kDa	0	5	1
MYO7A	254 kDa	0	0	3
YBX1	36 kDa	0	4	0
HNRNPA1	39 kDa	0	3	1
SRRM2	300 kDa	0	1	3
KIAA1107	156 kDa	0	0	3
ASH1L	333 kDa	0	0	3
CPSF6	59 kDa	0	4	0
EFCAB6	173 kDa	0	0	3
SECISBP2L	122 kDa	0	0	3
SRSF1	28 kDa	0	3	0
MIS188P1	129 kDa	0	0	3
MACF1	838 kDa	0	0	3

Supplementary Table 2. Hardy-Weinberg Equilibrium test for rs117701653 from gnomAD (v3.1.2).

	Observed genotype distribution			Expected genotype distribution			Chi-square	
Population	AA	AC	CC	AA	AC	CC	χ2	p value
European (non-Finnish)	30,833	3,092	86	30,825.3	3,107.4	78.3	0.83	0.66
African/ African American	20,186	516	6	20,183.4	521.3	3.4	2.12	0.35
Latino/ Admixed American	6,421	1,157	53	6,420.3	1,158.5	52.3	0.01	0.99
European (Finnish)	4,627	650	25	4,625.1	653.8	23.1	0.18	0.91
East Asian	1,963	598	39	1,967.9	588.1	43.9	0.73	0.69
South Asian	1,484	824	103	1,491.0	810.0	110.0	0.72	0.70
Ashkenazi Jewish	1,322	386	27	1,322.9	384.2	27.9	0.04	0.98
Other	904	132	9	900.4	139.2	5.4	2.82	0.24
Amish	453	3	0	453.0	3.0	0.0	0.00	1.00
Middle Eastern	125	29	4	123.2	32.7	2.2	1.99	0.37

Supplementary Table 3. Oligonucleotide sequences used for EMSA, qPCR, and CRISPP-Cas9

EMSA DNA fragment	Sequence
rs117701653-C/5Biosg	AATGATACGGCGACCACCGAGGATCC<u>GGTGTGTGCTTTGGGCGGATATGAGTGCACG</u> GAATTCTCGTATGCCGTCTTCTGCTTG
rs117701653-C	AATGATACGGCGACCACCGAGGATCC<u>GGTGTGTGCTTTGGGCGGATATGAGTGCACG</u>GAATTCTCGTATGCCGTCTTCTGCTTG
Irrelevant DNA	AATGATACGGCGACCACCGAGGATCC<u>AGGGCTGTAGATTCCGGCCTGAAGCCTGGG</u> GAATTCTCGTATGCCGTCTTCTGCTTG

ChIP-qPCR Primers	Sequence
HS17 Forward	CAAGGTCCATTACGCCACCT
HS17 Reverse	AGGGTTTGTCCCCAGGTACT
rs117701653 Forward	AGAAGGGGAGGAAGGTGTGT
rs117701653 Reverse	AGGGTATGCAAATGTTCCAG

rs117701653 editing by CRISPR-Cas9	Sequence
sgRNA	AGGAAGGUGUGUGCUUUGGGAGG
DNA Donor Template	ACTCACGTCCTTATTTAGGGTATGCAAATGTTCCAGTACACTCTCAAAGTGACAGATGTACTGTCCTATGTGTGTGCACTCATATCCGCCCAAAGCACACACCTTCCTCCCCTTCTCTCGAATGTTT

SMCHD1 KO by CRISPR-Cas9	Sequence
sgRNA	UACUGCUGUGGUUAUCACAGGGG

qPCR primer	Sequence	PrimerBank ID
β-Actin-Forward / Reverse	CATGTACGTTGCTATCCAGGC / CTCCTTAATGTCACGCACGAT	4501885a1
SMCHD1-Forward / Reverse	GCCACAGGACGGTGTACTTG / CCGACTGTAGCAGGTATAAGGTG	148839304c1
ICOS-Forward / Reverse	CAGGAGAAATCAATGGTTCTGCC / CCTTTTGTCTTAGTGAGATCGCA	251823951c1
CD28-Forward / Reverse	CTATTTCCCGGACCTTCTAAGCC / GCGGGGAGTCATGTTCATGTA	340545509c1
RAPH1-Forward / Reverse	CCAACTTTTCTTACCGCTTCTCC / ACTGTTTCCTGAACCAATGCTG	47132518c1

Supplementary Table 4. Lists of antibodies used in this study

T cell stimulation

Antigen	Supplier	Source	Clone	Catalog. No	Dilution	Application note by the manufacturer
CD3	Biolegend	Mouse	OKT3	317302	5 µg/ml	The OKT3 monoclonal antibody reacts with an epitope on the epsilon-subunit within the human CD3 complex
CD28	Biolegend	Mouse	CD28.2	302943	2 µg/ml	T cell costimulation
ICOS	Invitrogen	Mouse	ISA-3	16-9948-82	2 µg/ml	T cell activation

Western blotting

Antigen	Supplier	Source	Catalog. No	Dilution	Western blotting validation by the manufacturer
SMCHD1	ABCAM	Rabbit	ab179456	1:1000	293T, HepG2, HeLa, U-87 MG cell
alpha-Tubulin	Cellsignal	Rabbit	2144S	1:2000	CAD and C6 cell
ICOS	ABCAM	Rabbit	ab175401	1:1000	Jurkat and mouse thymus
RAPH1	Cellsignal	Rabbit	91138T	1:1000	MDA-MB-231, 293 293T, MCF7, HaCAT cell
CD28	Cellsignal	Rabbit	38774S	1:1000	RPMI 8226, Jurkat, human and mouse CD4+ T, RL-7, THP-1, EL4 cell
AKT	Cellsignal	Rabbit	9272S	1:1000	CHO, HeLa, NIH/3T3 cell
Ser473 Phospho-AKT	Cellsignal	Rabbit	4060S	1:1000	PC3, NIH/3T3 cell
JNK	Cellsignal	Rabbit	9252S	1:1000	293, SK-N-MC cell
Thr183/Tyr185 Phospho-JNK	Cellsignal	Rabbit	4668S	1:1000	293, NIH/3T3, C6

Immunophenotyping of CD4+ T cells subpopulations

Antigen	Conjujgate	Clone	Source	Supplier	Catalog. No	Dilution	Flow cytometry validation by the manufacturer
CD3	PE-Cy5	HIT3a	Mouse	Biolegend	300310	1:100	Human PBMC
CD4	BV605	OKT4	Mouse	Biolegend	317438	1:100	Human PBMC
CD4	BV786	OKT4	Mouse	Biolegend	317441	1:100	Human PBMC
CD25	BV786	BC96	Mouse	Biolegend	302638	1:100	Human PBMC
CD127	PE-Cy7	eBioRDR5	Mouse	Invitrogen	25-1278-42	1:100	Human PBMC
CD28	BV650	CD28.2	Mouse	Biolegend	302945	1:100	Human PBMC
CD45RA	BV711	HI100	Mouse	Biolegend	304137	1:100	Human PBMC
CD45RA	BV650	HI100	Mouse	Biolegend	304135	1:100	Human PBMC
CCR4	BV605	L291H4	Mouse	Biolegend	359417	1:100	Human PBMC
CCR6	PE	G034E3	Mouse	Biolegend	353409	1:100	Human PBMC
PD1	PE	EH12.2H7	Mouse	Biolegend	329905	1:100	Human PBMC
CXCR5	BV786	J252D4	Mouse	Biolegend	356935	1:100	Human PBMC
CXCR3	PE-Cy7	G025H7	Mouse	Biolegend	353719	1:100	Human PBMC
ICOS	APC/Cy7	C398.4A	menian Hams	Biolegend	313529	1:100	Human PBMC
FOXP3	APC	PCH101	Rat	Invitrogen	17-4776-42	1:100	Human PBMC

Antigen	Conjujgate	Clone	Source	Supplier	Catalog. No	Dilution
Isotype Control	PE-Cy5	MOPC-173	Mouse	Biolegend	400217	1:100
Isotype Control	BV605	MPC-11	Mouse	Biolegend	400349	1:100
Isotype Control	BV786	MPC-11	Mouse	Biolegend	400355	1:100
Isotype Control	BV786	MOPC-21	Mouse	Biolegend	400169	1:100
Isotype Control	PE-Cy7	MOPC-21	Mouse	Biolegend	400126	1:100
Isotype Control	BV650	MOPC-21	Mouse	Biolegend	400163	1:100
Isotype Control	BV711	MOPC-21	Mouse	Biolegend	400167	1:100
Isotype Control	BV650	MPC-11	Mouse	Biolegend	400351	1:100
Isotype Control	BV605	MOPC-21	Mouse	Biolegend	400161	1:100
Isotype Control	PE	eBMG2b	Mouse	Invitrogen	12-4732-81	1:100
Isotype Control	PE	P3.6.2.8.1	Mouse	Invitrogen	12-4714-81	1:100
Isotype Control	BV786	MOPC-21	Mouse	Biolegend	400169	1:100
Isotype Control	PE-Cy7	MOPC-21	Mouse	Biolegend	400126	1:100
Isotype Control	APC-Cy7	HTK888	Armenian Hamster	Biolegend	400927	1:100
Isotype Control	APC	eBR2a	Rat	Invitrogen	17-4321-81	1:100

In vitro Tph differentiation assay

Antigen	Conjujgate	Clone	Source	Supplier	Catalog. No	Dilution	Flow cytometry validation by the manufacturer
CD3	PE-Cy5	HIT3a	Mouse	Biolegend	300310	1:100	Human PBMC
CD4	BV605	OKT4	Mouse	Biolegend	317438	1:100	Human PBMC
CXCR5	BV786	J252D4	Mouse	Biolegend	356935	1:100	Human PBMC
PD1	PE	EH12.2H7	Mouse	Biolegend	329905	1:100	Human PBMC
CXCL13	AF700	NA	Mouse	R&D	IC801N	1:100	Human dendritic cells
IL-21	APC	3A3-N2		Biolegend	513007	1:100	Human PBMC

Antigen	Conjujgate	Clone	Source	Supplier	Catalog. No	Dilution
Isotype Control	PE-Cy5	MOPC-173	Mouse	Biolegend	400217	1:100
Isotype Control	BV605	MPC-11	Mouse	Biolegend	400349	1:100
Isotype Control	BV786	MOPC-21	Mouse	Biolegend	400169	1:100
Isotype Control	PE	P3.6.2.8.1	Mouse	Invitrogen	12-4714-81	1:100
Isotype Control	AF700	MOPC-21	Mouse	Biolegend	400143	1:100
Isotype Control	APC	MOPC-21	Mouse	Biolegend	400121	1:100
