# Supplementary material for: Non-coding autoimmune risk variant defines role for ICOS in T peripheral helper cell development
Source: Nat Commun. 2024 Mar 9;15:2150. doi: 10.1038/s41467-024-46457-8 (PMC10923805; doi:10.1038/s41467-024-46457-8)
Supplement: Supplementary file 3 — Reporting Summary [file 41467_2024_46457_MOESM3_ESM.pdf]

nature portfolio

Corresponding author(s): Peter A. Nigrovic
Last updated by author(s): YYYY-MM-DD

Reporting Summary

Nature Portfolio wishes to improve the reproducibility of the work that we publish. This form provides structure for consistency and transparency in reporting. For further information on Nature Portfolio policies, see our Editorial Policies and the Editorial Policy Checklist.

Statistics

For all statistical analyses, confirm that the following items are present in the figure legend, table legend, main text, or Methods section.

n/a	Confirmed	
☐	☒	The exact sample size (*n*) for each experimental group/condition, given as a discrete number and unit of measurement
☐	☒	A statement on whether measurements were taken from distinct samples or whether the same sample was measured repeatedly
☐	☒	The statistical test(s) used AND whether they are one- or two-sided<br>*Only common tests should be described solely by name; describe more complex techniques in the Methods section.*
☐	☒	A description of all covariates tested
☐	☒	A description of any assumptions or corrections, such as tests of normality and adjustment for multiple comparisons
☐	☒	A full description of the statistical parameters including central tendency (e.g. means) or other basic estimates (e.g. regression coefficient) AND variation (e.g. standard deviation) or associated estimates of uncertainty (e.g. confidence intervals)
☐	☒	For null hypothesis testing, the test statistic (e.g. *F*, *t*, *r*) with confidence intervals, effect sizes, degrees of freedom and *P* value noted<br>*Give P values as exact values whenever suitable.*
☒	☐	For Bayesian analysis, information on the choice of priors and Markov chain Monte Carlo settings
☒	☐	For hierarchical and complex designs, identification of the appropriate level for tests and full reporting of outcomes
☐	☒	Estimates of effect sizes (e.g. Cohen's *d*, Pearson's *r*), indicating how they were calculated

Our web collection on statistics for biologists contains articles on many of the points above.

Software and code

Policy information about availability of computer code

Data collection	CD3+CD4+ T cells from PBMCs of healthy subjects from the Mass General Brigham Biobank genotyped for rs117701653 (A/A = 8, A/C = 8, C/C = 8) were isolated by negative selection using EasySep human CD4+ T cell Isolation kit (STEMCELL, 17952). From the isolated CD3+CD4+ T cells of each individual, RNA was isolated using RNeasy Micro kit and eluted in 14 µl of water. 10 ng samples of RNA were transferred into wells of a 96-well plate, and RNA-seq libraries were prepared at Broad Technology Labs at the Broad Institute of MIT and Harvard (Cambridge, Massachusetts, USA) using the Illumina SmartSeq2 platform. Samples were sequenced on a NextSeq 500 generating a median of 5.6 million 38 bp paired-end reads per sample.
Data analysis	Raw data were processed using release 3.9 of the nextflow nf-core "rnaseq" pipeline. The pipeline was executed on the BCH HPC Clusters Enkefalos 2, using singularity containers to ensure optimal reproducibility. We performed adapter and quality trimming using Trim Galore (version 0.6.7) and subsequently aligned reads to the GRCh38 reference genome using STAR59 (version 2.7.10). We quantified transcript expression with Salmon60 (version 1.5.2) and aggregated transcript abundances to gene level measurements with bioconductor-tximeta (version 1.8.0). There is no use of commercial, open code, or custom code in this study.

For manuscripts utilizing custom algorithms or software that are central to the research but not yet described in published literature, software must be made available to editors and reviewers. We strongly encourage code deposition in a community repository (e.g. GitHub). See the Nature Portfolio guidelines for submitting code & software for further information.

Data

Policy information about availability of data

All manuscripts must include a data availability statement. This statement should provide the following information, where applicable:

- Accession codes, unique identifiers, or web links for publicly available datasets
- A description of any restrictions on data availability
- For clinical datasets or third party data, please ensure that the statement adheres to our policy

The Human reference genome utilized in this study was sourced from the genome assembly GRCh38 (https://www.ncbi.nlm.nih.gov/datasets/genome/GCF_000001405.40/). The genotype rs117701653 across known populations was retrieved from gnomAD genomes v3.1.2 (https://gnomad.broadinstitute.org/variant/2-203764072-A-C?dataset=gnomad_r3). Source data are provided with this paper. All data supporting the findings of this study are available within the paper and its Supplementary Information. Raw data for each panel may also be accessed through Nigrovic, Peter (2023), "2023Kim-ICOS-Tph", Mendeley Data, https://data.mendeley.com/datasets/7263bjmtxd/1). Gene expression matrices are accessible in NCBI's Gene Expression Omnibus via GEO Series accession number GSE235868 (https://www.ncbi.nlm.nih.gov/geo/query/acc.cgi?acc=GSE235868). Raw sequence data are available through dbGAP at phs003448.v1.p1. (http://www.ncbi.nlm.nih.gov/projects/gapprev/gap/cgi-bin/preview1.cgi?GAP_phs_code=IMZVjj7e4iUUV5vQ).

Research involving human participants, their data, or biological material

Policy information about studies with human participants or human data. See also policy information about sex, gender (identity/presentation), and sexual orientation and race, ethnicity and racism.

Reporting on sex and gender	This study involved 63 genotyped subjects, including 22 males and 41 females by self-declared sex. Subjects also included 8 healthy donors and 9 patients with RA, for whom demographic information was not provided. Sex was not considered in the recruitment of subjects or the choice of cell lines for in vitro manipulation.
Reporting on race, ethnicity, or other socially relevant groupings	In our research, we did not catergorize or analyze participant based on any socially constructed or socially relevant variables.
Population characteristics	Healthy subjects aged between 25 and 76 with23 AA, 23 AC, and 17 CC genotypes for SNP rs117701653 were recruited, excluding individuals who diagnosed with autoimmune and/or immune-mediated diseases. Patients with RA fulfilled the ACR/EULAR 2010 Rheumatoid Arthritis classification criteria
Recruitment	63 healthy participants were recruited from genotyped volunteer donors within the Mass General Brigham Biobank through the recruitment center of the Joint Biology Consortium (www.jbcwebportal.org). Each participant was compensated with a gift-card, and their sex was determined based on self-report.<br>Synovial fluid samples were obtained as excess material from 9 RA patients during clinically indicated diagnostic or therapeutic arthrocentesis procedures as directed by the treating rheumatologist.<br>Our studies were not powered to detect sex-based differences. This difference could primarily be due to the rarity of the C/C genotype. Instead, we addressed sex differences through statistically appropriate correction methods.
Ethics oversight	This study was approved by the Institutional Review Board (IRB) at Mass General Brigham, and all healthy participants provided informed consent. Blood samples from healthy controls were obtained from blood bank leukoreduction collars without written consent and were not employted for genomic studies.

Note that full information on the approval of the study protocol must also be provided in the manuscript.

Field-specific reporting

Please select the one below that is the best fit for your research. If you are not sure, read the appropriate sections before making your selection.

☒ Life sciences ☐ Behavioural & social sciences ☐ Ecological, evolutionary & environmental sciences

For a reference copy of the document with all sections, see nature.com/documents/nr-reporting-summary-flat.pdf

Life sciences study design

All studies must disclose on these points even when the disclosure is negative.

Sample size	For RNA sequencing to analyze eQTL of SNP rs117701653, we aimed to recruit a total of 24 samples, including 8 individuals with each of the A/A, A/C, and C/C genotypes. This balanced distribution was chosen to enhance the statistical power of the eQTL analysis, which was estimated over 90 % using the calculation tool available at (https://bwhbioinfo.shinyapps.io/powerEQTL/).<br><br>For immunophenotyping and differentiation assays using flow cytometry, we collected a total of 46 samples, comprising 17 A/A, 18 A/C, and 11 C/C donors. With a sample size of 46, we estimated that we would have 90 % statistical power for linear regression analysis between genotype groups, assuming a large effect size (https://www.statskingdom.com/33test_power_regression.html). Flow cytometry analysis was performed using PBMCs from blood samples of 8 healthy controls and synovial fluid samples of 9 RA patients. The sample size for each experiment was specified in the corresponding figure legend and method section.

Data exclusions	For mass cytometry analysis in Figure 4C, one sample from the RA patients was excluded as a marked outlier.
Replication	FREP and mass spectrometry was performed once.<br>FREP and Western blot was successfully confirmed with three replicates using distinct samples.<br>EMSA was successfully confirmed with three replicates using distinct nuclear extracts.<br>ChIP-qPCR with PBMC was performed once using a combination of four biological replicates.<br>For ChIP-qPCR, RNA-based qPCR, and western blot with CRISPR-edited clones, 13 biological replicates including 7 A/A, 3 A/C, and 3 C/C genotypes were tested once.<br>Association analysis between SNP rs117701653 with ICOS expression and Tph cell frequency by flow cytometry were tested once using 46 biological replicates including 17 A/A, 18 A/C, and 11 C/C genotypes.<br>Flow cytometry using PBMCs from blood 8 healthy donors and synovial fluid of 9 RA patients was performed once.<br>Tph differentiation assay with 6 healthy donors was performed once.<br>Tph differentiation assay with 46 donors including 17 A/A, 18 A/C, and 11 C/C genotypes was tested once.<br>SMCHD1 deletion in primary T cells was successfully confirmed with three replicates using distinct samples.<br>The effect of SMCHD1 deletion in T cells from 6 A/A and 5 C/C donors was tested once.
Randomization	To assess the association of genotype with ICOS expression and Tph differentiation, human samples were separated into groups based on their genotype for the SNP rs117701653. In order to remove the impact of age and sex as confounding factors, all analyses with human samples were adjusted for these demographic variables. In contrast, for all other experiment, human samples were randomly selected.
Blinding	This study focused on assessing the impact of genetic variation at SNP rs117701653 on a pathway regulating ICOS and Tph abundance. To achieve this, we recruited 46 healthy subjects based on their genotype from the MGB Biobank donors. The analysis of the experiment results involved grouping subjects by the genotype. Due to this specific approach, blinding was not applicable.<br>In contrast , for other experiments using human samples, blinding was implemented. Subjects were randomly selected to investigate the effect of ICOS on driving Tph differentiation.

Reporting for specific materials, systems and methods

We require information from authors about some types of materials, experimental systems and methods used in many studies. Here, indicate whether each material, system or method listed is relevant to your study. If you are not sure if a list item applies to your research, read the appropriate section before selecting a response.

Materials & experimental systems

n/a	Involved in the study	
☐	☒	Antibodies
☐	☒	Eukaryotic cell lines
☒	☐	Palaeontology and archaeology
☒	☐	Animals and other organisms
☒	☐	Clinical data
☒	☐	Dual use research of concern
☒	☐	Plants

Methods

n/a	Involved in the study	
☒	☐	ChIP-seq
☐	☒	Flow cytometry
☒	☐	MRI-based neuroimaging

Antibodies

Antibodies used	A list of antibodies for T cell stimulation:<br>Antigen/Supplier/Source/Clone/Catalog. No/Dilution/Application note by the manufacturer<br>CD3 Biolegend Mouse OKT3 317302 5µg/ml The OKT3 monoclonal antibody reacts with an epitope on the epsilon-subunit within the human CD3 complex<br>CD28 Biolegend Mouse CD28.2 302943 2µg/ml T cell costimulation<br>ICOS Invitrogen Mouse ISA-3 16-9948-82 2µg/ml T cell activation<br><br>A list of antibodies for Western blotting:<br>Antigen/Supplier/Source/Catalog. No/Dilution/Western blotting validation by the manufacturer<br>SMCHD1 ABCAM Rabbit ab179456 1:1000 293T, HepG2, HeLa, U-87 MG cell<br>alpha-Tubulin Cellsignal Rabbit 2144S 1:2000 CAD and C6 cell<br>ICOS ABCAM Rabbit ab175401 1:1000 Jurkat and mouse thymus<br>RAPH1 Cellsignal Rabbit 91138T 1:1000 MDA-MB-231, 293 293T, MCF7, HaCAT cell<br>CD28 Cellsignal Rabbit 38774S 1:1000 RPMI 8226, Jurkat, human and mouse CD4+ T, RL-7, THP-1, EL4 cell<br>AKT Cellsignal Rabbit 9272S 1:1000 CHO, HeLa, NIH/3T3 cell<br>Ser473 Phospho-AKT Cellsignal Rabbit 4060S 1:1000 PC3, NIH/3T3 cell<br>JNK Cellsignal Rabbit 9252S 1:1000 293, SK-N-MC cell<br>Thr183/Tyr185 Phospho-JNK Cellsignal Rabbit 4668S 1:1000 293, NIH/3T3, C6<br><br>A list of antibodies for Immunophenotyping of CD4+ T cells subpopulations:<br>Antigen/Conjujgate/Clone/Source/Supplier/Catalog. No/Dilution/Flow cytometry validation by the manufacturer<br>CD3 PE-Cy5 HIT3a Mouse Biolegend 300310 1:100 Human PBMC<br>CD4 BV605 OKT4 Mouse Biolegend 317438 1:100 Human PBMC<br>CD4 BV786 OKT4 Mouse Biolegend 317441 1:100 Human PBMC

CD25 BV786 BC96 Mouse Biolegend 302638 1:100 Human PBMC
CD127 PE-Cy7 eBioRDR5 Mouse Invitrogen 25-1278-42 1:100 Human PBMC
CD28 BV650 CD28.2 Mouse Biolegend 302945 1:100 Human PBMC
CD45RA BV711 HI100 Mouse Biolegend 304137 1:100 Human PBMC
CD45RA BV650 HI100 Mouse Biolegend 304135 1:100 Human PBMC
CCR4 BV605 L291H4 Mouse Biolegend 359417 1:100 Human PBMC
CCR6 PE G034E3 Mouse Biolegend 353409 1:100 Human PBMC
PD1 PE EH12.2H7 Mouse Biolegend 329905 1:100 Human PBMC
CXCR5 BV786 J252D4 Mouse Biolegend 356935 1:100 Human PBMC
CXCR3 PE-Cy7 G025H7 Mouse Biolegend 353719 1:100 Human PBMC
ICOS APC/Cy7 C398.4A Armenian Hamster Biolegend 313529 1:100 Human PBMC
FOXP3 APC PCH101 Rat Invitrogen 17-4776-42 1:100 Human PBMC
Antigen/Conjujgate/Clone/Source/Supplier/Catalog. No/Dilution
Isotype Control PE-Cy5 MOPC-173 Mouse Biolegend 400217 1:100
Isotype Control BV605 MPC-11 Mouse Biolegend 400349 1:100
Isotype Control BV786 MPC-11 Mouse Biolegend 400355 1:100
Isotype Control BV786 MOPC-21 Mouse Biolegend 400169 1:100
Isotype Control PE-Cy7 MOPC-21 Mouse Biolegend 400126 1:100
Isotype Control BV650 MOPC-21 Mouse Biolegend 400163 1:100
Isotype Control BV711 MOPC-21 Mouse Biolegend 400167 1:100
Isotype Control BV650 MPC-11 Mouse Biolegend 400351 1:100
Isotype Control BV605 MOPC-21 Mouse Biolegend 400161 1:100
Isotype Control PE eBMG2b Mouse Invitrogen 12-4732-81 1:100
Isotype Control PE P3.6.2.8.1 Mouse Invitrogen 12-4714-81 1:100
Isotype Control BV786 MOPC-21 Mouse Biolegend 400169 1:100
Isotype Control PE-Cy7 MOPC-21 Mouse Biolegend 400126 1:100
Isotype Control APC-Cy7 HTK888 Armenian Hamster Biolegend 400927 1:100
Isotype Control APC eBR2a Rat Invitrogen 17-4321-81 1:100

A list of antibodies for in vitro Tph differentiation assay:
Antigen/Conjujgate/Clone/Source/Supplier/Catalog. No/Dilution/Flow cytometry validation by the manufacturer
CD3 PE-Cy5 HIT3a Mouse Biolegend 300310 1:100 Human PBMC
CD4 BV605 OKT4 Mouse Biolegend 317438 1:100 Human PBMC
CXCR5 BV786 J252D4 Mouse Biolegend 356935 1:100 Human PBMC
PD1 PE EH12.2H7 Mouse Biolegend 329905 1:100 Human PBMC
CXCL13 AF700 NA Mouse R&D IC801N 1:100 Human dendritic cells
IL-21 APC 3A3-N2 Biolegend 513007 1:100 Human PBMC
Antigen/Conjujgate/Clone/Source/Supplier/Catalog. No/Dilution
Isotype Control PE-Cy5 MOPC-173 Mouse Biolegend 400217 1:100
Isotype Control BV605 MPC-11 Mouse Biolegend 400349 1:100
Isotype Control BV786 MOPC-21 Mouse Biolegend 400169 1:100
Isotype Control PE P3.6.2.8.1 Mouse Invitrogen 12-4714-81 1:100
Isotype Control AF700 MOPC-21 Mouse Biolegend 400143 1:100
Isotype Control APC MOPC-21 Mouse Biolegend 400121 1:100

Validation

Information on the application and validation of each primary antibody is described in the above section.

Eukaryotic cell lines

Policy information about cell lines and Sex and Gender in Research

Cell line source(s)

Jurkat T cells from ATCC

Authentication

The cell line used was not authenticated.

Mycoplasma contamination

The cell line was not tested for mycoplasma contamination.

Commonly misidentified lines
(See ICLAC register)

Jurkat is not a commonly misidentified cell line.

Flow Cytometry

Plots

Confirm that:

☒ The axis labels state the marker and fluorochrome used (e.g. CD4-FITC).

☒ The axis scales are clearly visible. Include numbers along axes only for bottom left plot of group (a 'group' is an analysis of identical markers).

☒ All plots are contour plots with outliers or pseudocolor plots.

☒ A numerical value for number of cells or percentage (with statistics) is provided.

Methodology

Sample preparation	PBMCs were isolated from blood via Ficoll gradient, and memory CD4+ T cells were further isolated from PBMCs using the EasySep kit for negative selection of human memory CD4+ T cells.
Instrument	We used the BD LSRFortessa cell analyzer for flow cytometry.
Software	The flow cytometer was operated using the BD FACSDiva software and the flow cytometry data was analyzed using Flowjo software version 10.8.0.
Cell population abundance	We measured the purity of isolated memory T cells using flow cytometry. More than 95 % were found to be CD3+CD4 +CD45RA- memory T cells.
Gating strategy	The positive region was identified by gating based on its fluorescence signal being greater than the background signal set by the negative isotype control.<br>We characterized major populations of human CD4+ T cells as follows: naive T cells (CD3+CD4+CD45RA+), memory T cells (CD3+CD4+ CD45RA-), and effector memory subsets: Th1 (CD3+CD4+CD45RA- CCR6-CXCR3+CCR4-), Th2 (CD3+CD4 +CD45RACCR6-<br>CXCR3-CCR4+), Th17 (CD3+CD4+CD45RA-CCR6+CXCR3-CCR4+), memory Treg (CD3+CD4+CD45RA-CD25highCD127-FOXP3+), Tfh (CD3+CD4+CD45RA-CXCR5+PD-Thigh), and Tph (CD3+CD4+CD45RA-CXCR5-PD-Thigh) (Supplementary Fig. 4).

☒ Tick this box to confirm that a figure exemplifying the gating strategy is provided in the Supplementary Information.
